# Enhancing Cannabinoid Bioavailability in Pain Management: The Role of Cyclodextrins

**DOI:** 10.3390/molecules29225340

**Published:** 2024-11-13

**Authors:** Adriana Ribeiro, Rui Loureiro, Helena Cabral-Marques

**Affiliations:** Research Institute for Medicines (iMed.ULisboa), Faculty of Pharmacy, Universidade de Lisboa, 1649-003 Lisbon, Portugal; ribeiroadriana@edu.ulisboa.pt (A.R.); loureiro@ff.ulisboa.pt (R.L.)

**Keywords:** chronic pain, cannabinoid, *Cannabis*, cyclodextrin, drug delivery, bioavailability, inclusion complexes, terpene, analgesic, anti-inflammatory

## Abstract

Chronic pain (CP), including pain related to cancer, affects approximately 2 billion people worldwide, significantly diminishing quality of life and imposing socio-economic burdens. Current treatments often provide limited relief and may cause adverse effects, demanding more effective alternatives. Natural compounds from *Cannabis sativa* L., particularly cannabinoids like THC and CBD, exhibit analgesic and anti-inflammatory properties, but their therapeutic use is restricted by poor solubility and low bioavailability. Cyclodextrins (CDs) and cyclic oligosaccharides may encapsulate hydrophobic drugs in order to enhance their solubility and stability, offering a promising solution to these challenges. This study explores the formation of CD inclusion complexes with cannabinoids and specific terpenes, such as D-limonene (LIM), beta-caryophyllene (BCP), and gamma-terpinene (γ-TPN), aiming to improve pharmacokinetic profiles and therapeutic efficacy. We discuss analytical techniques for characterizing these complexes and their mechanisms of action, highlighting the potential of CDs to optimize drug formulations. The integration of CDs in cannabinoid therapies may enhance patient compliance and treatment outcomes in CP management. Future research should focus on innovative formulations and delivery systems to maximize the clinical applications of those compounds.

## 1. Introduction

Pain is a significant concern in modern healthcare, defined by the International Association for the Study of Pain (IASP) as an unpleasant sensory and emotional experience shaped by intricate biological, psychological, and social factors [1,2]. Pain is classified as acute or chronic and may occur either continuously or intermittently [1]. Chronic pain (CP) is particularly problematic, as it persists over extended periods and profoundly diminishes quality of life for both patients and their families [1,3].

Globally, CP affects an estimated 2 billion people, imposing considerable physical, emotional, and socio-economic burdens [3,4]. In Portugal, approximately one-third (37%) of patients attending primary care services experience CP, significantly impacting their overall quality of life, particularly with regard to psychological well-being, and can lead to difficulties in physical movement [3]. Managing CP remains challenging, with current therapies often delivering inconsistent outcomes, offering only modest relief, and frequently producing side effects that hinder long-term treatment [3,5].

Pain represents a significant challenge for cancer patients, with breakthrough pain occurring in 40% to 80% of those undergoing opioid treatment [6]. This situation, coupled with the development of tolerance of analgesics, highlights the urgent need for alternative strategies in managing cancer-related pain. Moreover, concerns about long-term opioid use in cancer survivors with CP closely resemble those in patients with chronic non-malignant pain. Both cases highlight the necessity for advanced therapeutic approaches that offer pain control without the drawbacks of opioid dependency [7]. Advances in personalized medicine have emphasized the need for more precise and effective therapeutic approaches with reduced adverse effects. In this context, natural compounds, particularly those derived from *Cannabis sativa* L., have gained considerable attention for their potent analgesic and anti-inflammatory properties. This includes their effects on conditions associated with infections, which can manifest in various ways depending on the type of pathogen and the site of infection [8,9,10,11]. However, despite their potential therapeutic benefits, the clinical application of these compounds remains limited due to inherent challenges such as poor aqueous solubility, chemical instability, and low bioavailability, all of which significantly impede their therapeutic efficacy and potential in medical practice.

A promising approach to overcoming these challenges is the use of macrocyclic compounds, such as cyclodextrins (CDs). Natural CDs, including α-CD, β-CD, and γ-CD, are formed by the enzymatic degradation of starch and were first isolated in 1891 [12,13]. Their cavities of varying sizes allow them to effectively encapsulate bioactive compounds with unfavorable physicochemical properties, as well as enhance patient compliance through taste-masking [14,15,16]. CDs improve the solubility and bioavailability of pharmaceutical formulations by encapsulating hydrophobic molecules, commonly referred to as guest molecules [12,13]. The first drug marketed as a CD complex was a prostaglandin, known for its role in pain and inflammation management, followed by piroxicam formulated with β-CD in 1988 [17,18]. Since then, the range of pharmaceutical products utilizing CDs has expanded significantly, now exceeding 120 active ingredients, primarily based on β-CD or its derivatives, such as Hydroxypropyl-β-CD (HP-β-CD) [13,17]. Additionally, CDs can be employed in the design of supramolecular structures, including CD-based nanosponges, which facilitate enhanced stability and the controlled release of encapsulated molecules [19,20,21]. Generally Recognized As Safe (GRAS) and biodegradable by both the Food and Drug Administration (FDA) and the European Medicines Agency (EMA) [22], CDs are extensively utilized in large-scale pharmaceutical and food development [23,24].

In addition to natural CDs, chemically modified derivatives, such as hydroxypropylated (-CH_2_CH(OH)CH_3_), methylated (-CH_3_), sulfated butyl (-CH_2_CH_2_CH_2_CH_2_SO_3_^−^), and perallylated (-CH_2_CH=CH_2_ or -C_3_H_5_) forms are developed to further optimize these properties by enhancing water solubility, stability, and reducing toxicity [25,26]. These modifications significantly broaden the applicability of CDs in pharmaceutical formulations, particularly in improving the bioavailability of poorly soluble and unstable drugs. As a result, modified CDs are essential tools in advanced drug delivery systems [25].

Additionally, active pharmaceutical ingredients (APIs) are rarely administered in their pure form; they are usually formulated with excipients and vehicles, ranging from simple solutions to advanced controlled-release systems [27]. Approximately 90% of new chemical entities developed by the pharmaceutical industry exhibit poor water solubility, presenting a significant challenge for formulation scientists, with 40% being practically insoluble in water [28,29]. Poor solubility can compromise drug bioavailability and therapeutic efficacy, often necessitating higher doses to achieve the desired therapeutic effect [28]. Beyond solubility, low permeability is also a critical factor that affects bioavailability, particularly for lipophilic compounds such as cannabinoids. Bioavailability, defined as the proportion of an API that reaches systemic circulation, is central to therapeutic efficacy [30]. While this may seem self-evident, it is essential to emphasize that a drug can only achieve its intended therapeutic effect if sufficient concentrations are reached at the target site within the body. Thus, addressing both solubility and permeability barriers is crucial, as these factors together influence absorption and bioavailability. Consequently, formulation strategies that improve both factors are essential to enhance the therapeutic effectiveness of cannabis-derived APIs. Therefore, the pharmaceutical formulator plays a vital role in developing formulations that optimize bioavailability, ensure consistent dosing, and ultimately improve therapeutic outcomes [27].

Given the challenges posed by the suboptimal physicochemical properties of natural compounds, particularly in enhancing bioavailability, this article focuses on the pharmaceutical development of the primary cannabinoids, tetrahydrocannabinol (THC) and cannabidiol (CBD). Their analgesic effects, especially in neuropathic and CP, have been approved for therapeutic use in several countries, including conditions like multiple sclerosis, where they help reduce spasticity [31].

In addition, the acidic precursors of these cannabinoids, along with terpenes from *C. sativa*, contribute significantly to their therapeutic profile. This study highlights the use of CD complexation, including both native and modified CDs, as a strategy to improve cannabinoids bioavailability. Furthermore, it discusses the analytical techniques used to characterize these inclusion complexes, which are essential for distinguishing encapsulated drugs from their free forms and evaluating bioavailability enhancement.

## 2. Formation of Host–Guest Inclusion Complexes with Cyclodextrins

**Figure 1:**
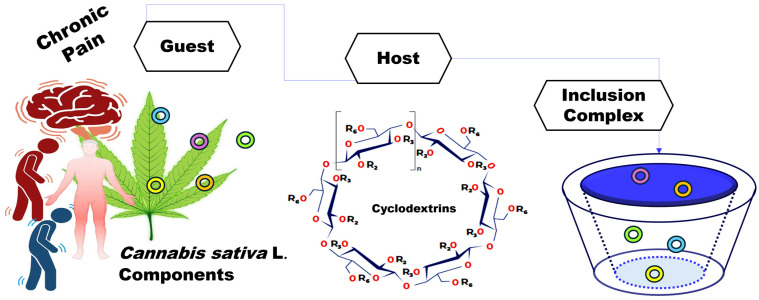
Formation of host–guest inclusion complexes between *C. sativa* components and CDs, illustrating the glucose unit count (*n*), along with substitution sites (R_2_, R_3_, R_6_) for creating CD derivatives. Natural CDs; all R substituents are hydrogen atoms.

An inclusion complex is formed when a “host” molecule, typically a CD, creates a cavity in its structure that allows it to accommodate a “guest” molecule—often a nonpolar substance or one with lower polarity than water, such as a drug. This process is driven by the release of water molecules from the hydrophobic cavity of the CD into the surrounding medium, which can lead to either an exothermic or endothermic enthalpy change, depending on the specific guest molecule. This inclusion mechanism involves enthalpy–entropy compensation, without forming or breaking covalent or ionic bonds [32,33]. Figure 1 visually illustrates this concept, where *C. sativa* components are the guest molecules encapsulated by CDs, forming stable inclusion complexes.

The formation of these complexes is influenced by several factors, including size, geometry, stereochemistry, polarity, and the presence of suitable binding sites. Variables such as temperature, co-solvents, and pH can modulate these interactions [32]. The toroidal structure of CDs, characterized by an internal hydrophobic cavity and an external hydrophilic surface due to the arrangement of hydroxyl groups on glucose units, allows for the partial or full insertion of the guest molecule. This encapsulation protects and stabilizes the guest, significantly enhancing its solubility and bioavailability [33]. Additionally, intermolecular forces, such as dipole–dipole interactions and van der Waals forces, contribute to the stabilization of the complex, making CDs versatile tools in pharmaceutical formulations [33,34].

**Figure 2:**
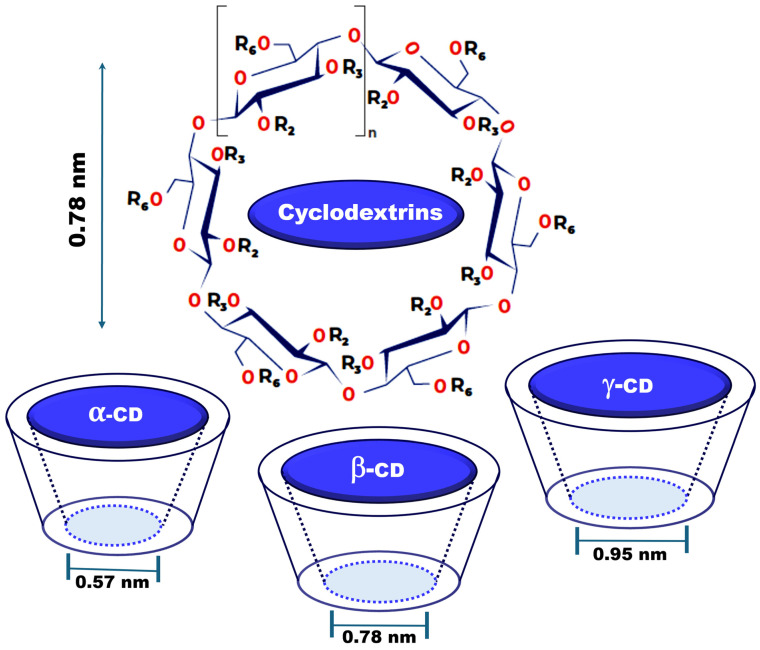
Structure and dimensions of natural CDs, where n = 1, 2, or 3 represents α-, β-, or γ-CDs, and R_2_, R_3_, and R_6_ can be substituted by different radicals to obtain CD-derivatives.

CDs are classified according to the number of glucose residues in their structures, giving rise to α-CD (hexamer), β-CD (heptamer), and γ-CD (octamer) forms, with cavities of approximately 0.6 nm, 0.8 nm, and 1 nm, respectively (Figure 2) [12,27].

Moreover, chemically modified derivatives of CDs have tailored properties that provide several advantages, such as HP-β-CD and Randomly Methylated-beta-cyclodextrin (RM-β-CD), have been developed to enhance solubility and stability, thereby increasing the efficacy of these complexes in specific pharmaceutical applications (Table 1).

Modified CDs, such as HP-β-CD and SBE-β-CD, have obtained regulatory approval in various jurisdictions, including the European Union, where they are listed in the European Pharmacopoeia, and in the United States, where they are classified as GRAS food additives [23,24]. In Japan, CDs are acknowledged as natural products and included in the Japanese Pharmacopoeia [39]. However, it is important to note that while CDs are generally considered safe, caution is advised regarding their use in certain populations and dosages. For example, the safe use of CDs in children under 2 years old has not been established, and products containing a threshold greater than 20 mg/kg/day should only be administered when the benefits outweigh the risks, according to a healthcare practitioner. Additionally, the oral administration of CDs at doses exceeding 200 mg/kg/day may lead to gastrointestinal disturbances, such as diarrhea and cecal enlargement. Some CDs, like α-CD, β-CD, and RM-β-CD, are not suitable for parenteral use due to renal toxicity, while γ-CD, HP-β-CD, and SBE-β-CD are regarded as relatively safe, although concentrations above 200 mg/kg/day for extended treatments warrant caution in patients with renal impairment due to the risk of accumulation [22,23,24].

**Figure 3:**
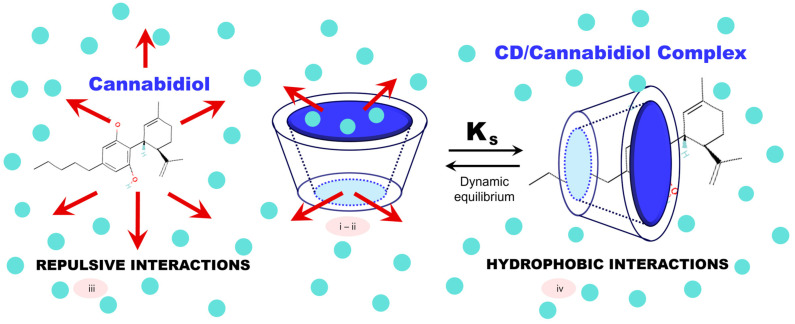
Inclusion complexes involving cannabidiol (CBD) and CDs. This process entails (i) the displacement of water molecules from the hydrophobic cavity of the CD, allowing room for CBD; (ii) increased hydrogen bonding between the displaced water and the surrounding medium; (iii) minimized repulsive interactions between CBD and the aqueous environment; and (iv) strengthened hydrophobic interactions as CBD is fully encapsulated within the CD cavity. This mechanism enhances CBD’s bioavailability and therapeutic potential by protecting it from degradation and improving its solubility.

The stoichiometry of inclusion complexes formed between CDs and guest molecules can vary, with possible arrangements such as 1:1, 1:2, 2:1, and 2:2 [40]. While the most common ratio is 1:1 (Figure 3), where one CD molecule complexes with one guest molecule (e.g., CBD), determining the exact stoichiometry can be challenging. Recent studies, such as the re-evaluation of the β-CD complex, have shown that initial assumptions about host ratios can sometimes persist for years until resolved by advanced analytical techniques [41].

Recent studies have highlighted the distinct entrapment mechanisms of CBD within the cavities of α-CD, β-CD, and γ-CD, resulting in the formation of host–guest complexes characterized by stoichiometries of 1:1, 2:1, and 2:1, respectively [42,43]. This complexation significantly enhances the aqueous solubility of CBD, with solubility levels increasing from 6.3 × 10^−5^ mg/mL to 3.7 mg/mL for α-CD, 2.1 mg/mL for β-CD, and 5.3 mg/mL for γ-CD [42,43].

These findings emphasize the necessity of meticulous characterization methods to accurately determine the structural configurations of these complexes. This is particularly critical as nanoformulations present a viable strategy to address the challenges associated with cannabinoid delivery [43].

### 2.1. Technological Advantages: Stability, Solubility, and Bioavailability

CD-based inclusion complexes provide several key technological advantages for the development of pharmaceutical formulations. These complexes effectively address the common challenges in drug formulation by enhancing the stability, solubility, and bioavailability of bioactive compounds [44].

One significant benefit is physicochemical stabilization, where inclusion complexes with CDs protect against thermal decomposition, oxidation, and hydrolysis. This is especially important for compounds like cannabinoids, which exhibit low stability; such protection ensures sustained efficacy over time.

Furthermore, the solubility and bioavailability of drugs are notably improved when complexed with CDs. According to the Biopharmaceutical Classification System (BCS, Table 2), a drug is considered soluble if the highest intended human dose dissolves in 250 mL of water, which represents the FDA’s “glass of water” standard. Low-water-solubility drugs, such as cannabinoids, can achieve enhanced absorption across biological barriers through this complexation, thereby facilitating delivery to target sites and improving their effectiveness in pain management [45].

In addition, inclusion complexes can be utilized to develop sustained-release systems, which allow for the prolonged administration of bioactive compounds like THC and CBD. This capability is particularly crucial for highly permeable drugs (Classes I and III of the BCS) in the treatment of chronic pain, where controlled release can optimize pharmacokinetic profiles and provide extended relief.

Moreover, encapsulation within CDs can mitigate unpleasant odors and tastes, enhancing patient compliance, especially in oral formulations. Selecting the most suitable CD—whether α-, β-, or γ-CD or one of their derivatives —for each specific compound is vital for optimizing the formation of inclusion complexes (best molecular adjustment) and achieving the best therapeutic outcomes. Additionally, CDs can convert liquids and oils into free-flowing powders and prevent incompatibilities in mixtures, thereby aligning with the solubility and permeability characteristics of drugs as defined by the BCS.

Beyond their technological advantages, the application of CDs can help overcome the limitations imposed by Lipinski’s “Rule of 5” and the BCS classifications, which are crucial in determining the effectiveness of oral drugs. Lipinski’s “Rule of 5” is a widely recognized principle in drug discovery that helps predict the similarity of a new compound to existing drugs based on five key physicochemical parameters: molecular mass, lipophilicity, polar surface area, hydrogen bond donors, and electric charge. These parameters are essential for optimizing the permeability of new compounds through passive diffusion. According to this rule, a compound is less likely to have absorption or permeation issues if it has fewer than five hydrogen bond donors, fewer than ten hydrogen bond acceptors, a molecular weight below 500 g/mol, and a Log P (lipophilic partition coefficient) value less than 5 [46,47,48].

For oral drugs, an ideal Log P is between 1.35 and 1.8 to ensure effective absorption in both the gastrointestinal tract and systemic circulation. However, it is important to note that Lipinski specified that the “Rule of 5” applies only to compounds that are not substrates of active transporters. Therefore, while the rule provides valuable guidance for the initial evaluation of new compounds, its applicability is limited to those that rely on passive transport mechanisms to cross biological membranes.

By forming inclusion complexes, CDs offer a way to circumvent the restrictions imposed by the “Rule of 5” and the BCS, allowing compounds that initially do not meet these criteria to be successfully developed as effective drugs.

### 2.2. Overview of Inclusion Complex Preparation Methods

The preparation of inclusion complexes with CDs is crucial for enhancing the solubility and bioavailability of poorly soluble compounds. Several methods have been developed, each with unique advantages that can be tailored to optimize the effectiveness of these complexes [49]. Table 3 summarizes the primary methods used for preparing inclusion complexes, highlighting their descriptions, advantages, and typical applications. Notably, the co-precipitation method is applicable only to natural CDs, as complexes formed with soluble CD derivatives do not precipitate effectively, limiting their use in certain formulations.

Each of these methods presents unique benefits that can significantly impact the development of effective pharmaceutical formulations. By selecting the appropriate preparation technique, researchers can enhance the stability, solubility, and overall therapeutic efficacy of drugs formulated with CDs. These methods have proven especially beneficial in improving the pharmacokinetic profiles of lipophilic and poorly soluble compounds, including cannabinoids such as THC, CBD, and other bioactive phytochemicals.

As noted previously, the formation of inclusion complexes with CDs represents a promising strategy to enhance the bioavailability of bioactive compounds, particularly those with poor solubility, which is a significant challenge in the management of pain and inflammation [56]. These complexes markedly improve the physicochemical properties and stability of drugs, thereby facilitating their incorporation into pharmaceutical formulations [23]. A notable application is the complexation of prostaglandins, such as PGE1 and PGE2, which exhibit low water solubility. The use of α- and β-CDs can enhance their solubility by up to 50-fold and significantly improve their thermal stability, increasing the stability of these compounds from 15% to 90% at 40 °C [57]. This enhancement is crucial for the therapeutic efficacy of prostaglandins, which play vital roles in pain modulation and inflammatory responses.

Furthermore, nonsteroidal anti-inflammatory drugs (NSAIDs) represent a category of medications commonly used for alleviating pain, reducing inflammation, and managing fever. These drugs generally contain organic compounds as their main active ingredients, including diclofenac [58]. The use of HP-β-CD has been shown to increase the solubility of diclofenac by up to five times, enabling the achievement of therapeutic concentrations with a reduced risk of adverse effects. Similarly, the solubility of dexamethasone can be enhanced by as much as 33-fold through β-CD complexation [17,59]. Such improvements in solubility are essential for optimizing the clinical effectiveness of analgesics and anti-inflammatory agents.

A variety of CDs employed in drug delivery systems targeting pain and inflammation have been well documented (Table 4), including approved drugs.

Notably, ketoprofen (KTP) and oxaprozin are under investigation for their potential benefits when complexed with CDs. Studies show that KTP forms a stable 1:1 inclusion complex with methyl-beta-cyclodextrin (M-β-CD), resulting in improved dissolution rates and enhanced bioavailability, potentially boosting its anti-inflammatory and anti-arthritic activities compared to KTP alone [21].

Similarly, oxaprozin’s solubility is being enhanced through combinations with RM-β-CD and L-arginine (ARG) or sepiolite nanoclay (SV). A quaternary nanocomposite comprising the drug–RM-β-CD-ARG complex entrapped in SV demonstrated an impressive increase in dissolution rate from 60% to 90% compared to ternary systems. In vivo studies on rats indicated that a fast-dissolving tablet formulation of this quaternary system provided a more rapid and potent pain-relieving effect in treating adjuvant-induced arthritis potential therapeutic advantages in formulations currently under evaluation [66].

### 2.3. Role in Pain Management

**Figure 4:**
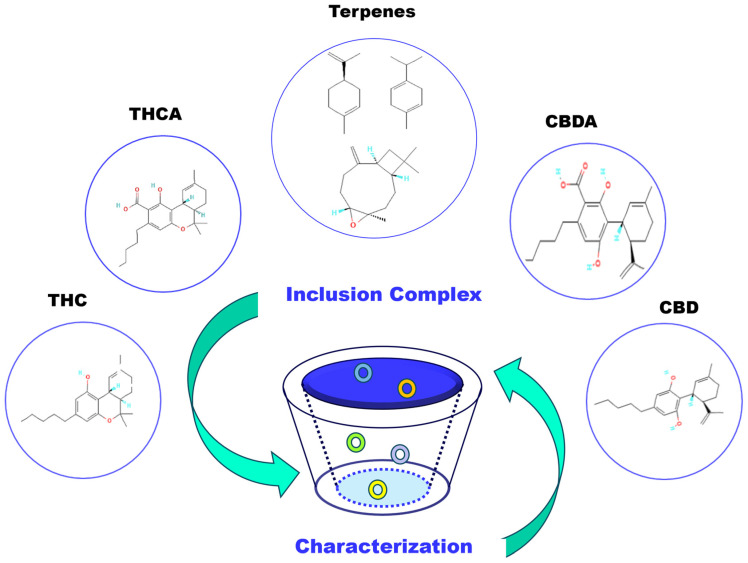
CD inclusion complexes’ formation with cannabinoids and terpenes, highlighting the characterization.

The following sections explore the significance of CDs in enhancing the bioavailability of cannabinoids and terpenes in pain management, with a focus on the formation of inclusion complexes. Additionally, the importance of characterizing these complexes is discussed, which is essential to ensure the efficacy and stability of the formulations (Figure 4).

#### 2.3.1. Cannabinoids: THC, CBD, and Their Acidic Precursors

*C. sativa* contains a variety of bioactive compounds, among which THC and CBD stand out as the most prominent phytocannabinoids, largely due to their extensive research and wide-ranging therapeutic applications.

Although they share the same molecular formula (C_21_H_30_O_2_), their distinct structures lead to markedly different interactions with the endocannabinoid system, which comprises endogenous cannabinoids such as anandamide (N-arachidonoylethanolamine-AEA) and 2-arachidonoylglycerol (2AG). These cannabinoids interact with cannabinoid receptors CB1 and CB2, which are G-protein coupled receptors (GPCRs) crucial for modulating various physiological processes, including pain perception and inflammation [9,67].

Among the cannabinoids, THC, with its bicyclic structure, has a high affinity for both cannabinoid receptors, CB1 and CB2. As a partial agonist of these receptors, THC is effective in modulating pain perception, inflammation, and other neurological processes, resulting in potent analgesic effects, particularly in CP conditions [68,69]. Additionally, THC influences the release of neurotransmitters such as dopamine and serotonin, which are regulated by enzymes like fatty acid amide hydrolase (FAAH) and monoacylglycerol lipase (MAGL) that degrade endocannabinoids. Notably, THC possesses an anti-inflammatory potency 20 times greater than that of aspirin and twice that of hydrocortisone, while avoiding the drawbacks associated with COX-1 or COX-2 inhibition at therapeutic concentrations. However, the activation of CB1 receptors by THC is also responsible for the psychoactive effects often associated with *C. sativa* use [10,70,71].

In contrast, CBD’s structural configuration prevents it from directly binding to CB1 and CB2 receptors, thereby avoiding psychoactive effects. Instead, CBD exerts its analgesic and anti-inflammatory effects through multiple pathways, including the modulation of serotonin receptors (5-HT1A), the activation of the transient receptor potential vanilloid 1 (TRPV1), and inhibition of anandamide degradation. These mechanisms allow CBD to provide pain relief, particularly in neuropathic pain and inflammation, without the adverse effects often associated with THC [69,71,72].

Beyond its role in pain relief, CBD also demonstrates significant antioxidant and anti-inflammatory effects. It reduces reactive oxygen species (ROS), tumor necrosis factor-alpha (TNF-α), and vascular endothelial growth factor (VEGF) expression. By preventing the formation of superoxide, the death of retinal cells, and vascular hyperpermeability, CBD helps to mitigate retinal inflammation [73]. Both THC and CBD exhibit antioxidant properties, enabling them to neutralize free radicals and protect against oxidative processes [74,75,76]. Furthermore, studies indicate that CBD can reduce hyperalgesia and improve thermal perception in animal models, underscoring its potential in treating inflammatory and neuropathic pain [10]. CBD also inhibits adenosine uptake in microglia and decreases the production of pro-inflammatory cytokines, further highlighting its therapeutic potential in inflammatory and neurodegenerative conditions [73]. Notably, a CBD extract was critical in the treatment of pain in patients with opioid-refractory cancer, demonstrating a 30% reduction in pain, whereas THC-rich extracts devoid of CBD did not show greater efficacy than a placebo [10].

Alongside THC and CBD, their acidic precursors, THCA (tetrahydrocannabinolic acid) and CBDA (cannabidiolic acid), have gained recognition for their anti-inflammatory and analgesic properties [77,78]. THCA, the non-psychoactive precursor of THC, exerts anti-inflammatory effects by inhibiting cyclooxygenase-2 (COX-2) and reducing inflammatory cytokines such as TNF-α. Similarly, CBDA interacts with TRPV1 receptors, involved in pain signaling, and inhibits COX-2, making it an effective anti-inflammatory agent. These precursors show potential for treating inflammatory and neuropathic pain without psychoactive effects [79].

The challenges facing all four cannabinoids—THC, CBD, THCA, and CBDA—include poor water solubility and low bioavailability. These limitations can be mitigated through inclusion complexes with CDs, which enhance their solubility, stability, and therapeutic efficacy. By improving bioavailability, CD complexation enables these cannabinoids, in both acidic and neutral forms, to offer more efficient and sustained relief from pain and inflammation, reducing side effects and dosing frequency.

#### 2.3.2. Enhancing Cannabinoid Bioavailability with Cyclodextrins

CDs offer a promising solution to the poor water solubility and bioavailability of cannabinoids such as THC and CBD, which hinder their effectiveness in pain management. Several studies have demonstrated that forming inclusion complexes with different CD isoforms, including α-CDs, β-CDs, and γ-CDs, significantly improves the solubility and stability of hydrophobic compounds, leading to enhanced absorption and clinical effectiveness.

For instance, β-CD, HP-β-CD, and RM-β-CD have been shown to not only improve the solubility of THC and CBD but also prolong their therapeutic effects, optimizing their pharmacokinetics and therapeutic outcomes [80,81,82]. Studies with β-CD inclusion complexes revealed improved anti-inflammatory and antinociceptive activities, demonstrating that CDs modulate the delivery time profile and enhance the therapeutic efficacy of cannabinoids in vivo [80,81,82,83].

Research indicates that CBD, when complexed with CDs (particularly HP-β-CD), demonstrates significantly enhanced solubility and bioavailability. This is critical in the context of anti-inflammatory activity, as CBD can inhibit the production of inflammatory cytokines such as IL-1β, IL-6, TNF-α, and IFN-β, which are implicated in chronic inflammation and cancer progression [84].

Studies involving β-CD inclusion complexes have shown that CBD complexed with β-CD demonstrates a superior anti-inflammatory effect compared to both pure CBD and ibuprofen, even at reduced doses. For instance, a dose of 4.375 mg/kg of CBD/β-CD achieved the same anti-inflammatory results as 8.75 mg/kg of pure CBD. Additionally, while ibuprofen alone was effective in reducing edema, its anti-inflammatory efficacy improved when complexed with β-CD (ED50, i.e., Effective Dose IBU = 30.5 mg/kg; ED50 IBUβ-CD = 24.3 mg/kg). This suggests that CDs can significantly enhance the therapeutic efficacy of both cannabinoids and conventional anti-inflammatory drugs [85].

Additionally, the incorporation of γ-CD into metal–organic frameworks (γ-CD-MOFs) represents an innovative approach to enhance the bioavailability of cannabinoids, such as olivetol—a compound known for its potential therapeutic properties and as a precursor to synthetic cannabinoids. These MOFs have shown remarkable capacity for encapsulating bioactive compounds, enabling controlled and sustained release, which is essential for effective pain and inflammation management. Studies indicate that using co-crystallization and impregnation methods significantly increases the olivetol loading in γ-CD-MOFs, highlighting their efficacy compared to traditional CD systems. The biocompatibility of γ-CD-MOFs, coupled with their structural stability, suggests substantial potential for clinical applications, effectively overcoming the limitations of conventional formulations [86].

In this area, another study with intracerebroventricular (ICV) administration of THC complexed with HP-β-CD (THC/HP-β-CD) has shown potent antinociceptive effects, like those observed with traditional solvents, while also mitigating safety concerns associated with those solvents, particularly for treating chronic pain [81]. Moreover, sublingual inclusion complexes of THC with RM-β-CD have demonstrated higher bioavailability compared to ethanolic THC solutions, as well as promoting faster absorption, resulting in a more efficient therapeutic response. In comparison to HP-β-CD, RM-β-CD showed better complexation efficiency with cannabinoids, leading to enhanced solubility and absorption rates [80].

Similarly, CBD inclusion complexes with CDs, particularly RM-β-CD, have increased solubility by up to 1000-fold, significantly improving absorption and prolonging therapeutic action [42]. By forming these inclusion complexes, CDs enhance the concentration gradient across cell membranes, facilitating better drug absorption. Studies indicate that CBD/β-CD complexes significantly increase plasma levels of CBD compared to conventional formulations, thereby enhancing its therapeutic efficacy in managing pain and inflammation [87]. Additionally, the sublingual administration of these complexes bypasses first-pass metabolism, further optimizing bioavailability [88].

In addition to THC and CBD, the CD inclusion complexes have shown significant promise for improving the pharmacokinetic properties of THCA and CBDA. For instance, when THCA is complexed with M-β-CD, its solubility and permeability are significantly enhanced under physiological conditions. This increased bioavailability allows THCA to exert its therapeutic effects, including its anti-inflammatory properties, more effectively and for longer durations, thereby improving its overall efficacy in managing pain and inflammation [79]. CBDA has similarly shown improved solubility and stability when complexed with RM-β-CD, enhancing its absorption and prolonging its therapeutic action. These improvements make CBDA more effective at delivering sustained relief from pain and inflammation, optimizing its use in clinical settings [79].

Overall, the bioavailability enhancements provided by CDs improve the absorption of THC, CBD, THCA, and CBDA but also enable more consistent and prolonged therapeutic effects [88,89]. Notably, CBD can be encapsulated within the hydrophobic central cavity of CDs, achieving solubility levels of up to 5000 μg/mL [90]. This reduces the need for frequent dosing and enhances patient adherence to pain and inflammation management protocols [88,89].

Moreover, recent advancements in cannabinoid delivery systems include formulations based on HP-βCD, which have been employed to enhance the bioavailability of MDA7, a lipophilic cannabinoid type 2 agonist.

Studies have demonstrated that MDA7 can be effectively encapsulated within HP-βCD, resulting in significantly improved solubility and pain-relieving effects that alleviate allodynia (an abnormal pain sensation) compared to traditional formulations. The inclusion complex formed between MDA7 and HP-βCD exhibits a 1:1 stoichiometry, indicating efficient encapsulation. In vivo experiments showed that HP-βCD-based formulations administered intravenously led to enhanced therapeutic responses, underscoring the potential of CD-based systems in optimizing the delivery of lipophilic cannabinoids. This highlights not only the versatility of CDs in cannabinoid applications but also their role in advancing formulations for pain management therapies [91].

#### 2.3.3. Limonene: A Monoterpene with Analgesic Potential

D-limonene (LIM), a cyclic monoterpene present in citrus oils and specific *Cannabis* strains, is well known for its analgesic and anti-inflammatory effects. Studies indicate that LIM is effective in reducing neuropathic and inflammatory pain, with clinical trials confirming its low toxicity and favorable safety profile even with long-term use. Despite these benefits, LIM’s hydrophobic characteristics complicate its delivery and bioavailability [10,92].

To enhance its therapeutic potential, LIM can be complexed with β-CD. Research demonstrates that this LIM-β-CD complex exhibits significant antinociceptive effects at all assessed time points (*p* < 0.001). Notably, pre-treatment with naloxone did not reverse its analgesic activity, suggesting an alternative mechanism of action. A two-way ANOVA analysis demonstrated significant effects of LIM treatment over time, with an F-value of 15.4 (degrees of freedom: 5 and 60) and a *p*-value of less than 0.0001. The complex significantly increased paw withdrawal thresholds for up to eight hours post-treatment, in contrast to uncomplexed LIM, which was effective for only four hours (*p* < 0.001). Additionally, treatment with LIM-β-CD resulted in a significant increase in Fos-positive cells in the spinal cord (F [2, 15] = 53.9; *p* < 0.001), indicating enhanced analgesic efficacy. These findings underscore the potential of LIM-β-CD complexes as a promising strategy for managing chronic musculoskeletal pain and improving patient outcomes [92].

#### 2.3.4. Beta-Caryophyllene (BCP): A Potent Anti-Inflammatory Terpene

BCP synthesized by *C. sativa* is a sesquiterpene composed of 15 carbon atoms (C_15_H_24_), found in various plants such as clove (*Syzygium aromaticum*), black pepper (*Piper nigrum*), and rosemary (*Rosmarinus officinalis*) [10,93,94]. Among the different terpenes produced by *C. sativa*, BCP is one of the most prevalent and significantly contributes to the spicy and woody aroma of the plant. BCP is characterized by an unusual cyclobutane ring, which distinguishes it from other terpenes and imparts unique properties [93].

In the realm of pharmacology, BCP selectively activates the CB2 cannabinoid receptors, primarily located in immune cells and peripheral tissues. This selective action is crucial for modulating inflammation and pain while avoiding the psychoactive effects associated with CB1 receptor activation [10,95,96]. BCP has demonstrated significant efficacy in reducing mechanical and cold hypersensitivity. Notably, its effects can be enhanced when combined with CBD, resulting in additive effects in males and synergistic effects across both sexes [97].

Moreover, BCP exhibits remarkable anti-inflammatory effects that are particularly beneficial for managing CP. BCP seems to be a potent natural supplement capable of suppressing hepatic inflammation and carcinoma through the mitigation of oxidative stress and inflammatory pathways [98]. By engaging CB2 receptors, BCP reduces levels of pro-inflammatory cytokines, including TNF-α, interleukins (IL-1β, and IL-6). These cytokines are known to exacerbate inflammation and increase pain perception, making BCP especially advantageous for conditions such as arthritis, where chronic inflammation plays a significant role in pain [96].

In addition to its anti-inflammatory properties, BCP demonstrates a direct analgesic action by influencing pain signal transmission in peripheral nerves. Its interaction with CB2 receptors helps to diminish pain signaling pathways, making it effective in treating neuropathic pain, which often resists conventional treatments [95]. Importantly, this analgesic effect is achieved without activating CB1 receptors, allowing BCP to alleviate pain without the psychoactive effects linked to THC [97].

Furthermore, the synergistic antinociceptive effect of combining paracetamol with BCP could be advantageous for the management of inflammatory pain, and the gastroprotective activity should help to protect against the adverse effects of chronic use [99]. Recent research has indicated that combining BCP with β-CD significantly improves its bioavailability and therapeutic efficacy. In a mouse model of chronic muscle pain, BCP-β-CD complexes effectively reduced mechanical hyperalgesia and enhanced muscle withdrawal thresholds, all while maintaining normal motor function. Furthermore, this treatment decreased Fos protein expression in the superficial dorsal horn of the spinal cord, suggesting a central role in pain modulation [94].

Overall, these insights suggest that BCP, particularly when paired with CBD and β-CD, could represent a valuable therapeutic strategy for managing chronic pain. This combination offers both anti-inflammatory and analgesic benefits, alongside improved bioavailability and safety.

#### 2.3.5. Gamma-Terpinene (γ-TPN): Cancer Pain Management

The monoterpene γ-TPN found in *C. sativa* oil is noted for its high lipophilicity, which restricts its solubility and systemic absorption; this characteristic can limit its therapeutic efficacy in managing chronic pain, particularly in cancer patients who often experience intense neuropathic pain.

A promising approach to overcome these limitations is the complexation of γ-TPN with β-CD, resulting in the γ-TPN/β-CD complex. Experimental studies conducted in sarcoma 180 models demonstrated that the administration of this complex not only alleviated hyperalgesia but also reduced inflammatory markers such as TNF-α and IL-1β in tumor tissue. Additionally, β-CD prolonged the analgesic effects of γ-TPN, indicating that the formation of the complex stabilizes the compound and enhances its duration of action. This combination demonstrated a significant reduction in inducible Nitric Oxide Synthase (iNOS) activity and c-Fos protein expression in the spinal cord, both associated with pain signaling and inflammation [100]. Results showed a significant decrease (*p* < 0.001) in mechanical hyperalgesia following daily treatment for six days with γ-TPN (50 mg/kg, p.o.) and γ-TPN/β-CD (50 mg/kg, p.o.). Assessments using the Grip and Rota-rod techniques indicated no interference with muscle strength and motor coordination in the animals, suggesting that the doses used do not exert depressant effects on the central nervous system [101].

A notable characteristic of γ-TPN is its interaction with voltage-dependent calcium channels. Studies using patch-clamp techniques showed that γ-TPN reduces calcium (Ca^2+^) currents, suggesting an effective mechanism for pain modulation. Molecular docking analyses indicated favorable interactions between γ-TPN and these channels, which may explain its ability to attenuate neuronal activity and reduce pain perception [100].

Utilizing β-CD to encapsulate γ-TPN not only improves the solubility and stability of the compound but also enhances its therapeutic properties. This innovative approach allows γ-TPN to act more effectively in cancer pain management, presenting itself as a viable alternative in contexts where conventional treatment options fall short. Thus, the combination of γ-TPN with β-CD represents a valuable strategy for optimizing the management of pain associated with cancer, promoting both anti-inflammatory and analgesic effects [100].

The clinical implications of these findings are substantial, underscoring the therapeutic potential of the γ-TPN/β-CD complex as a novel and effective approach for managing neuropathic pain in cancer patients. This complex not only enhances analgesic efficacy but also offers an improved safety profile, which is particularly important for patients who often experience adverse effects from conventional therapies.

In summary, CD complex formation has proven effective in enhancing the solubility, stability, and bioavailability of cannabinoids and terpenes, particularly in the context of pain management, as demonstrated in various studies.

#### 2.3.6. Structural and Physicochemical Properties: Cannabinoid and Terpene Inclusion Complexes

Table 5 presents a diverse array of inclusion complexes formed with CDs and highlights significant advancements, along with their respective characterization methods and formulation potential.

The characterization of inclusion complexes is essential to prove complex formation and thus ensure the efficacy and stability of pharmaceutical formulations. β-CD plays a crucial role in forming inclusion complexes, significantly improving the solubility and stability of bioactive compounds like LIM. For instance, Differential Scanning Calorimetry (DSC) revealed that the slurry complex (SC) of LIM and β-CD demonstrated reduced volatilization and enhanced thermal stability compared to free LIM and β-CD. This improvement highlights the potential of β-CD to increase the bioavailability of hydrophobic compounds, making them more effective in therapeutic applications [92].

In the study of γ-TPN and its complex with β-CD, in vivo and in silico methods were used to explore the therapeutic interactions and properties of the inclusion complex. These methods confirmed the formation of the complex and provided insights into its physicochemical properties, including thermal stability and molecular interactions. Docking studies suggested favorable interactions between γ-TPN and various receptors (e.g., adrenergic alpha-2, glutamatergic, opioid, and cholinergic), indicating the complex’s potential mechanism of action in managing pain and inflammation. The combination of DSC, Fourier Transform Infrared Spectroscopy (FTIR), and Thermogravimetry (TGA) helped assess the stability and interactions within the complex, while X-ray Diffraction (XRD) confirmed changes in the crystallinity of β-CD following the inclusion process [100].

Moreover, inclusion complexes of cannabinoids—such as THC and CBD—with CDs have demonstrated significant improvements in solubility and bioavailability. Complexation with RM-β-CD significantly increased the dissolution rates of both cannabinoids, with times of 3.2 min for CBD and 3.6 min for THC, compared to 29.5 and 9.4 min for the pure forms, respectively. In vivo pharmacokinetic studies in rabbits showed that the sublingual bioavailability of the THC/RM-β-CD complex was 12.1%, which is higher than the bioavailability of ethanolic THC given orally (4.0%) and the sublingual bioavailability of ethanolic THC (3.8%). The time to reach maximum concentration was 120 min, indicating that sublingual administration not only enhances bioavailability but also accelerates the therapeutic effect [80]. Studies involving γ-CD and CBD complexes showed notable thermal stability, with a reduced decomposition rate compared to non-encapsulated compounds. These findings suggest that CDs can protect cannabinoids from degradation, prolonging their therapeutic effects [86].

Additionally, XRD has been proven useful in evaluating the crystallinity of inclusion complexes, with results showing that the inclusion of lipophilic compounds like THC and CBD in CDs reduces crystallinity, favoring solubility [79,85]. For instance, the crystallinity peaks of ibuprofen differed significantly from those of CBD, highlighting the technique’s ability to distinguish between the two drugs in their pure forms [85]. This structural change is critical for enhancing drug delivery systems in the management of pain and inflammation [79].

Scanning Electron Microscopy (SEM) also provides valuable insights into the morphology of particles after complexation. For instance, the β-CD/BCP complexes revealed more homogeneous and granular structures compared to the uncomplexed BCP, confirming the successful formation of the inclusion complex [94]. This change highlights the improvement in the structural characteristics following complexation.

Nuclear Magnetic Resonance (NMR) spectroscopy is crucial for elucidating the interactions between cannabinoids, such as CBD, and cyclodextrins, particularly in enhancing solubility and bioavailability. The encapsulation of CBD within the hydrophobic cavity of β-CD leads to significant alterations in NMR signals, reflecting changes in the chemical environment of the CBD protons. Specifically, protons associated with the alkyl side chains of CBD exhibit upfield shifts, indicating effective encapsulation within the β-CD structure. Detailed spectral analysis facilitates the determination of complex stoichiometry and insights into host–guest dynamics, as evidenced by the disappearance of peaks in the aromatic region indicative of cannabinoid immobilization [85].

^1^H NMR studies reveal that the inclusion of cannabinoid acids within the cavities of cyclodextrins (CDs) results in substantial chemical shift changes, indicating dynamic interactions between the functional groups of cannabinoids and the glucopyranose units of CDs. Notable shifts in the signals of protons H-3 and H-5 suggest the insertion of hydrophobic side chains of cannabinoids into the cavity of M-β-CD, indicating a stable configuration for the complex. The identification of distinct peaks in the 2.4–2.7 ppm region, attributed to aromatic methyl groups, reinforces the specificity of these interactions, which depend on the molecular conformation of the cannabinoids [79].

Furthermore, Mass Spectrometry (MS) is employed to determine the molecular mass and structural integrity of inclusion complexes, ensuring that the bioactive compound is not degraded during encapsulation. Techniques such NMR, involving THC complexes, and 2D NMR (NOESY/ROESY) confirmed the encapsulation of THC inside the RM-β-CD cavity, highlighting the interactions between the THC and the CD protons [102], and consequently the space-relative position of both molecules.

For example, in studies of THC and MDA7 inclusion complexes, other spectroscopic techniques were used to determine the stoichiometry and stability of the complexes. The THC-β-CD complex, for instance, achieved a THC concentration of 14 mg/mL with a 2:1 binding ratio, providing enhanced solubility for clinical applications [102]. Similarly, the inclusion of MDA7 in HP-β-CD resulted in the formation of a 1:1 complex, further improving the bioavailability and antinociceptive effects of the drug in pain management [91].

Overall, natural β-CDs are widely used in pharmaceutical applications; their utility stems not only from their appropriately sized cavities to ensure a close fitting but also from their ease of production and affordability. Although β-CDs have some limitations, such as inclusion capacity, solubility, and, in certain cases, toxicity, numerous β-CD derivatives have been developed to overcome these challenges [27,103]. These derivatives improve the solubility profile and safety, enhancing the efficacy of inclusion complexes with hydrophobic compounds, such as cannabinoids and terpenes. Ongoing innovations in CD-based drug delivery systems offer promising avenues for more effective therapies, positively influencing pain management and enhancing patient adherence to treatment.

### 2.4. Conclusions and Outlook

This study highlights the potential of CDs in enhancing the bioavailability and therapeutic efficacy of compounds derived from *C. sativa*, including key cannabinoids such as THC and CBD, as well as terpenes like limonene (LIM), beta-caryophyllene (BCP), and gamma-terpinene (γ-TPN). Studies have demonstrated that the formation of inclusion complexes with CDs significantly increases the solubility, stability, and absorption of these compounds, resulting in prolonged and improved therapeutic effects in pain and inflammation management.

Moreover, CDs have proven effective in modulating the pharmacokinetic profiles of these compounds, possibly promoting controlled and sustained release that optimizes clinical response, particularly in chronic and neuropathic pain conditions. The utilization of CDs, including β-CD, RM-β-CD, and HP-β-CD, not only enhances the bioavailability of cannabinoids and terpenes but also promises to increase treatment safety by mitigating potential adverse effects associated with traditional formulations.

The bioactive compounds present in *C. sativa* play a significant role in suppressing inflammatory processes through various mechanisms. They inhibit the production of pro-inflammatory cytokines, reduce the activity of enzymes involved in inflammation, block transcription factors related to the inflammatory response, and interfere with kinase signaling pathways, which are crucial for regulating inflammatory responses. CDs offer a novel way to improve the therapeutic potential of these bioactive compounds by overcoming their limitations in solubility and bioavailability.

Furthermore, advancements in CD-based formulations, including innovative metal–organic frameworks (MOFs), present promising opportunities for the development of new drug delivery systems, expanding the clinical applications of these compounds. These findings provide a solid foundation for ongoing investigations into the application of CDs in improving cannabinoid and terpene therapies, particularly in challenging treatment areas such as inflammatory, neuropathic, and cancer-related pain.

In summary, CDs represent an effective and versatile strategy for overcoming the limitations associated with the suboptimal physicochemical properties of cannabinoids and terpenes, optimizing their clinical use and promoting better therapeutic outcomes. Future research should focus on exploring the long-term effects of CD complexation in clinical settings, as well as investigating novel formulations that can further enhance the therapeutic potential of these compounds.

## Figures and Tables

**Table 1:** Key chemically modified cyclodextrins and their pharmaceutical applications.

Modified CD	Key Properties	Common Applications	Regulatory Status	Ref.
HP-β-CD	▪ Improved solubility and stability.	▪ Widely used to enhance the bioavailability of poorly soluble drugs.	▪ Approved for pharmaceutical use. EU and USA as excipients.	[35]
RM-β-CD	▪ Increased solubility and complexation ability.	▪ Often used in drug delivery systems to improve drug absorption.	▪ Limited regulatory approval; primarily in research.	[36]
SBE-β-CD	▪ Enhanced solubility and reduced toxicity.	▪ Commonly used in injectable formulations.	▪ Approved for pharmaceutical use. EU and USA as excipients.	[35]
CM-β-CD	▪ Enhanced water solubility and potential for targeted delivery.	▪ Used in formulations requiring specific pH conditions.	▪ Primarily in research; not widely approved.	[37]
Ac-β-CD	▪ Improved interaction with hydrophobic drugs.	▪ Useful in stabilizing volatile or unstable compounds.	▪ Mainly used in research; limited approval.	[38]

Modified CD: Ac-β-CD_Acetyl-beta-cyclodextrin; CM-β-CD_Carboxymethyl-beta-cyclodextrin; HP-β-CD_Hydroxypropyl-beta-cyclodextrin; RM-β-CD_Randomly Methylated-beta-cyclodextrin; SBE-β-CD_Sulfobutylether-beta-cyclodextrin.

**Table 2:** Biopharmaceutical Classification of Drugs. Adapted from [27].

Class	Solubility	Permeability	Oral Absorption Pattern	Limitation in Oral Absorption
I	High	High	Good absorption	Gastric emptying
II	Low	High	Variable	Dissolution
III	High	Low	Variable	Permeability
IV	Low	Low	Low absorption	Case-by-case

**Table 3:** Common methods for preparing inclusion complexes with cyclodextrins.

Method	Description	Advantages	Applications	Ref.
Co-precipitation	▪ Mixing natural CD and drug in solution, followed by precipitation.	▪ Simple process, good yield.	▪ Used for various natural compounds to enhance solubility.	[27,50]
Kneading	▪ Mixing CD and drug with a minimal amount of water to form a paste under slight heat and pressure.	▪ Solvent-minimized process that improves thermal stability.	▪ Effective for volatile compounds and improving stability.	[27,50]
Supercritical Carbon Dioxide	▪ Using carbon dioxide in a supercritical state to solubilize and encapsulate the drug.	▪ Produces high-purity products with no solvent residues.	▪ Ideal for encapsulating lipophilic and volatile compounds.	[51,52]
Grinding	▪ Reducing particle size through joint grinding of drug and CD.	▪ Simple and solvent-free, enhancing uniformity.	▪ Improves oxidation protection and formulation consistency.	[53,54]
Microwave Irradiation	▪ Using microwave energy to heat and facilitate the formation of inclusion complexes.	▪ Rapid and efficient, promoting higher retention of compounds.	▪ Useful for expediting complex formation.	[53,54]
Spray Drying	▪ Atomizing a solution of CDs and drugs, followed by rapid drying to form powder.	▪ Suitable for large-scale production with fine particles.	▪ Commonly used in pharmaceutical formulations to improve solubility.	[51,55]
Freeze Drying	▪ Dissolving the drug in water, mixing with the CD under agitation, and freeze-drying the mixture under reduced pressure.	▪ Suitable for water-soluble drugs; yields high-quality powder.	▪ Ideal for thermolabile drugs; forms powders with good yield.	[27,51]

Note: Co-precipitation is applicable only to natural CDs, as complexes involving soluble CD derivatives do not precipitate effectively.

**Table 4:** Marketed drug–CD complexes globally related to pain and inflammation.

CD Type	APIs	Formulation	Therapeutic Class	Ref.
ᾳ-CD	PGE1	Intravenous	Pain Mediator	[17,18]
β-CD	Aceclofenac	Oral	NSAID	[17,60]
β-CD	Dexamethasone	Oral	Corticosteroid	[17,18]
β-CD	Diclofenac	Parental, ocular	NSAID	[17,61]
β-CD	Flurbiprofen	Buccal	NSAID	[17,62]
β-CD	Ibuprofen	Oral	NSAID	[17,63]
β-CD	Meloxicam	Oral, rectal	NSAID	[17,64]
β-CD	Methyl salicylate	Dermal	Analgesic; AI	[17,18]
β-CD	Nimesulide	Oral	NSAID	[17,18]
β-CD	Paracetamol	Oral, buccal	Analgesic; AP	[17,18]
β-CD	PGE2	Sublingual	Pain Mediator	[17,18]
β-CD	Piroxicam	Oral	NSAID	[17,65]
β-CD	Rofecoxib	Oral	NSAID	[17,18]
β-CD	Tiaprofenic acid	Oral	NSAID	[17,18]
γ-CD	Curcumin extract	Oral	NAI	[17,18]
HP-β-CD	Diclofenac	Parenteral, ocular	NSAID	[17,18]
HP-β-CD	Flurbiprofen	Buccal	NSAID	[17,18]
HP-β-CD	Indomethacin	Ocular	NSAID	[17,18]
HP-β-CD	Paracetamol	Parenteral, buccal	Analgesic; AP	[17,18]
HP-γ-CD	Diclofenac	Ocular	NSAID	[17,18]

CD_Cyclodextrins: HP-β-CD_Hydroxypropyl β-Cyclodextrin; HP-γ-CD_Hydroxypropyl γ-Cyclodextrin. APIs_Active Pharmaceutical Ingredients: PGE1_Prostaglandin E1; PGE2_Prostaglandin E2. Therapeutic Class: AI_Anti-inflammatory; AP_Antipyretic; NAI_Natural Anti-inflammatory; NSAIDs_Non-Steroidal Anti-Inflammatory Drugs.

**Table 5:** Cyclodextrin inclusion complexes of cannabinoids and terpenes.

Inclusion Complex	Characterization Method							Experimental Model	Potential Formulation	Ref.
	DSC	TGA	FTIR	XRD	SEM	NMR	MD			
β-CD/CBD	✓	✓	✓	✓		✓		▪ in vivo	Oral	[85]
β-CD/BCP	✓	✓	✓	✓	✓			▪ in vivo	Oral	[94]
β-CD/γ-TPN							✓	▪ in vivo	Oral	[100]
γ-CD/MOFs	✓	✓	✓	✓	✓			▪ in vitro	Oral	[86]
RM-β-CD/THC		✓				✓		▪ in vitro	Oral	[102]
RM-β-CD/THC								▪ in vivo	Sublingual	[80]
M-β-CD/THCA	✓	✓	✓	✓	✓	✓		▪ in vitro	Oral	[79]
M-β-CD/CBDA	✓	✓	✓	✓	✓	✓		▪ in vitro	Oral	[79]
HP-β-CD/CBD	✓	✓	✓	✓	✓			▪ in vivo	Oral	[84]
HP-β-CD/THC			✓					▪ in vivo	Intracerebroventricular	[81]
HP-β-CD/MDA7	✓	✓	✓					▪ in vivo	Intravenous	[91]
HP-β-CD/LIM	✓	✓	✓					▪ in vitro	Oral	[92]

Abbreviations: CBDA_Cannabidiolic acid; CBD_Cannabidiol; THCA_Tetrahydrocannabinolic acid; THC_Tetrahidrocanabinol; BCP_Beta-caryophyllene; γ-TPN_Gamma-terpinene.; DSC_Differential scanning calorimetry; TGA_Thermogravimetry/derivative thermogravimetry; FTIR_Fourier Transform Infrared Spectrophotometer; XRD_X-ray diffraction; SEM_Scanning electron microscopy; NMR_Nuclear Magnetic Resonance; MD_Molecular docking.

## Data Availability

Data are unavailable due to privacy.
